# Case of Strangulated Hernia

**Published:** 1874-09

**Authors:** J. W. Robbins

**Affiliations:** Manchester, Iowa


Article IV.—*Case of Strangulated Hernia, Reduced by Gravitation and Traction.* By J. W. Robbins, M.D., Manchester, Iowa.

On the 8th ult. had a call to Mr. Sellens, aged 85 years. Has had hernia for many years. Says it would always go back itself, till this time, and now it had been down a week, and he could not get it back, and it was becoming very troublesome and painful.

On examination, I found a direct, intestinal hernia protruding from the internal ring of the left side, feeling to the touch hard as stone.

Having ascertained that the bowels were in a favorable condition, I gave him morphia and ipecac, and placed a sack of wet salt over the tumor. After waiting half an hour, I attempted reduction by taxis, and, failing, as I expected to, I then placed the legs of the patient over the shoulders of an assistant, and had his body raised nearly to a perpendicular position. Then, while manipulating the tumor with one hand, I grasped the abdomen with the other, and made traction as well as I could. To the joy of all concerned, the tumor, with the characteristic gurgle, suddenly disappeared.

Describing the case to a medical friend, he thought the best way was to get the finger into the ring, and then it could be reduced very readily. In my case, I think one might about as easily get the finger into an auger-hole with a wooden pin in it.

